# The best method of hepatitis B vaccination in hemodialysis patients?

**DOI:** 10.12861/jrip.2013.40

**Published:** 2013-10-10

**Authors:** Ali Momeni

**Affiliations:** ^1^Division of Nephrology, Department of Internal Medicine, Shahrekord University of Medical Sciences, Shahrekord, Iran

**Keywords:** Vaccination, Hepatitis B, Hemodialysis

**Implication for health policy/practice/research/medical education:**

After a long time, the efficacy of vaccination against hepatitis B could be decreased and thus for maintenance of protective antibody titer, booster dose of vaccine is required. It seems that additional studies with different dose of vaccine, duration and method are necessary for finding of the best method of vaccination in terms of safety, effectiveness and convenience application of vaccine among hemodialysis patients.

Hepatitis B is the most important cause of cirrhosis in many countries. Hemodialysis patients are susceptible to hepatitis due to repeated contact with dialysis machines and blood products (1). For immunization against hepatitis B, intramuscular (deltoid) vaccination in months 0, 1 and 6 is the standard method in normal population; and after immunization, HBS antibody titer should be greater than 10 mIU/mL (2). Approximately 90-95% of healthy people and 45-50% of dialysis patients properly respond to vaccination (3). In dialysis patients, although, usual method of vaccination is double dose of recombinant vaccine in months 0, 1, 2 and 6; due to insufficient response and rapid decline on antibody titer, different methods of vaccination were used in hemodialysis patients. In a cross-sectional study in hemodialysis patients of Hajar Hospital of Shahrekord, Iran, 24 cases of 78 hemodialysis patients without any appropriate response to conventional method of vaccination (double dose in 0, 1 and 6 months) and antibody titer less than 10 mIU/mL, were randomly assigned to intramuscular and intradermal groups. In intradermal (ID) group, 10 µg (0.5 ml) recombinant vaccine was prescribed every 2 weeks to 6 months, but in intramuscular (IM) group, 40 µg (2 ml) at 0, 1, 2 and 6 months were prescribed. At the beginning of the study, mean HBsAg specific antibody titer in patients was 4.4±3.1 mu/ml and after 1 month and 3 months, mean HBsAg antibody titer was 190.4 ± 59 mIU/mL and 223.3 ± 83.9 mIU/mL, respectively (p< 0.001). In both groups, the antibody titers were checked after 1, 3, 14 and 18 months after the last vaccination. Eight patients excluded from study because of transplantation, death or uncooperation. Furthermore, after 14 and 18 months, HBS antibody titer was rechecked in 16 remaining patients. Mean antibody titer in 14^th^ and 20^th^ month was 144±64 mIU/mL and 45±34ug/ml, respectively. There was no significant difference between antibody titer in two groups in all stages of the study. We found that the antibody titer decreased significantly over time, although at the end of the study, antibody titer was slightly more than minimum of protective titer (greater than 10 Iu/ml). However, other studies reported different and occasionally controversial results. For example, in Morais’ study, patients with antibody titer less than 10 mu/ml were received low dose of intradermal HB vaccine; and later in 82% of patients, antibody titer were greater than 10 mIU/mL after 12 monthes of vaccination (4). In Sorkhi’s study, after 6 months of vaccination, low dose intradermal and subcutaneous vaccination resulted in less seroconversion versus intramuscular method (5). Similar to our results, Somboonsilp showed that in end-stage renal disease ( ESRD) patients, there were no significant difference in seroconversion rates (anti HBs levels above 10 mIU/mL) between the intradermal and intramuscular groups at months 1, 2 and 7 (6). As a result, in majority of studies, after a long time, the efficacy of vaccination could be decreased and thus for maintenance of protective antibody titer, booster dose of vaccine is required. It seems that additional studies with different dose of vaccine, duration and method are necessary for finding of the best method of vaccination in terms of safety, effectiveness and convenience application of vaccine among hemodialysis patients.

## Author’s contribution

AM is the single author of the manuscript.

## Conflict of interests

The author declared no competing interests.

## Ethical considerations

Ethical issues (including plagiarism, data fabrication, double publication) have been completely observed by the author.

## Funding/Support

None.
